# TB Control in Humans and Animals in South Africa: A Perspective on Problems and Successes

**DOI:** 10.3389/fvets.2018.00298

**Published:** 2018-11-27

**Authors:** Christina Meiring, Paul D. van Helden, Wynand J. Goosen

**Affiliations:** Division of Molecular Biology and Human Genetics, Faculty of Medicine and Health Sciences, DST-NRF Centre of Excellence for Biomedical Tuberculosis Research, South African Medical Research Council Centre for Tuberculosis Research, Stellenbosch University, Cape Town, South Africa

**Keywords:** tuberculosis, *Mycobacterium bovis*, bovine TB, infectious diseases, zoonotic TB

## Abstract

*Mycobacterium tuberculosis* (*M. tb*) remains one of the most globally serious infectious agents for human morbidity and mortality, but with significant differences in prevalence across the globe. In many countries, the incidence is now low and declining, but control and eradication remain a distant view. Similarly, the prevalence of bovine TB caused by *Mycobacterium bovis (M. bovis)*, varies significantly across regions, although unlike for *M. tuberculosis*, data are sparse. The reduction in incidence and prevalence and control of both human and bovine TB is difficult and costly, yet some countries have managed to do this with some success. This perspective will consider some of the critical control steps we now know to be important for the control of TB from *M. tuberculosis* in humans living in South Africa, where the incidence of TB is the highest currently experienced. Despite the high incidence of human TB, South Africa has been able to reduce this incidence remarkably in the past few years, despite limited resources and high HIV prevalence. We draw from our experience to ascertain whether we may learn useful lessons from control efforts for both diseases in order to suggest effective control measures for bovine TB.

## Introduction

*Mycobacterium bovis (M. bovis)*, the causative agent of bovine tuberculosis (BTB), has perhaps the broadest host range of the pathogenic mycobacteria (1). Although the most commonly affected species are members of the Bovidae, even humans can be affected.

Considerably more attention is devoted to control of *Mycobacterium tuberculosis* in humans, than *M. bovis* in its multiple hosts (2). Although there are some similarities between TB control in humans and animals, such as the need for diagnosis, there are also very different disease management options, such as antibiotic therapy for humans, in comparison to test and slaughter for domestic cattle. Disease control measures include the need to find and deal with cases and prevent transmission. Although this seems self-evident, achieving these goals is not simple and require critical activities such as those shown below and discussed later.

Steps to TB control:

1. Awareness
2. Risk factor reduction
3. Access
4. Diagnosis
5. Retention
6. Treatment
7. Adherence
8. Follow up

Actions attributable to these steps allowed South Africa to steadily reduce human TB incidence from a peak of 977/100,000 per annum in 2007 to 781 in 2016. This observed reduction in incidence is perhaps remarkable because the reduction alone exceeds by far the incidence rate seen in most countries (3).

The reported occurrence of bovine TB in South African domestic bovine herds is far lower (Table 1), although since full testing coverage is not done the actual numbers are likely to be higher. TB and BTB control activities will be discussed below.

**Table 1:** *Mycobacterium bovis* cases reported in South Africa from 2000 to 2018 (Department of Agriculture, Forestry and Fisheries: http://www.daff.gov.za/daffweb3/Branches/Agricultural-Production-Health-Food-Safety/Animal-Health/Epidemiology).

**Year**	**Outbreaks**	**Cases**	**Dead/Culled**
2000	10	174	181
2001	1	33	1
2002	4	123	32
2003	17	394	370
2004	11	1,525	737
2005	14	747	856
2006	4	42	37
2007	6	102	50
2008	4	50	37
2009	18	36	1,236
2010	8	18	7
2011	7	34	29
2012	3	90	0
2013	2	8	29
2014	8	102	66
2015	8	32	28
2016	3	247	0
2017	1	8	0
2018	3	4	3

*Estimated cattle herd size 13.5 million in 2003*.

## Awareness and stigma

Ignorance of TB is rife. For this reason, many organizations tasked with human health care such as WHO (World Health Organization), The Union (International Union Against Tuberculosis and Lung Disease), and MSF (Médecins Sans Frontières), start their campaigns with generating awareness. Such campaigns leverage media, to create interest and awareness. Our own academic department has reached out to schools and communities in multiple activities in 2018 alone. Using past and cured patients to propagate the message through their own experiences can be quite effective at community level. Such public activities have the benefit of addressing and reducing stigma that might be attached to TB. There is now improved awareness amongst the South African public concerning human TB. However, there is little awareness of bovine TB. In general, there has not been much media attention, there is no large or even small-scale campaign, no rallying cry, no catch phrases, and essentially it is left to private and state veterinarians and technicians to work with farmers as they see fit. To date, one awareness day has been organized in only one location, and the limitations of this hardly need to be discussed.

## Risk factor reduction

Humans and animals share some common risk factors for TB, such as nutrition or malnutrition, age, crowding, and extent of exposure (4). There are many others which are likely to be restricted to humans or animals only, such as substance abuse in humans and environmental contamination in animals. Many risk factors in humans relate to poverty and are very difficult to address. Risk factors for cattle include historical TB on a farm, movement of animals, TB on neighboring property or in wildlife in contact with domestic stock, prevalence of TB in a herd or area and herd size, multiple premises, poor housing, and nutrition (5). It is often possible to mitigate against these risks for livestock.

A cornerstone of bovine TB control is movement restriction of animals. This is a vital activity, which is not generally possible with humans and therefore presents veterinarians with an enormous advantage to prevent ongoing disease transmission. Most countries have a test and slaughter policy in place for bovine TB in domestic stock (6, 7). However, having a policy and program does not necessarily mean that full coverage is achieved and appropriate action is followed. For example, many resource-poor countries such as South Africa do not have the resources for rigorous testing and there is a lack of compensation to affected livestock owners. Movement restriction requires proper monitoring, which is extremely difficult even under optimal circumstances. Although TB does not have a vector, we can argue that a contaminated environment (soil, water) and multiple hosts may act as reservoirs for infection and therefore also need active management.

## Access

In order to capitalize on awareness campaigns, it is vital that access to appropriate facilities and experts are available to persons who are ill. In South Africa, there is a large network of state-funded public health clinics (8) and private practitioners which addresses health problems including TB. In the veterinary field, there is a network of state veterinary services as well as private veterinarians who can deal with bovine TB. However, the veterinary service is far smaller than the human health service component and overall they must deal with far larger numbers of potential hosts on a per capita basis than clinicians for the human population. Testing for bovine TB is voluntary, except for dairy herds. However, there are inadequate numbers of state veterinarians to do regular TB testing, including for dairy herds where compulsory testing every 2 years is required. Therefore, private veterinarians have to be hired at considerable cost to the owners. On occasion, state veterinary services will provide TB testing for impecunious owners or commonage herds. Since there is no compensation paid to owners for culled positive animals or herds that need to be slaughtered, there is little or no incentive for testing to be done, in fact, there can be active resistance to testing.

**Figure 1:**
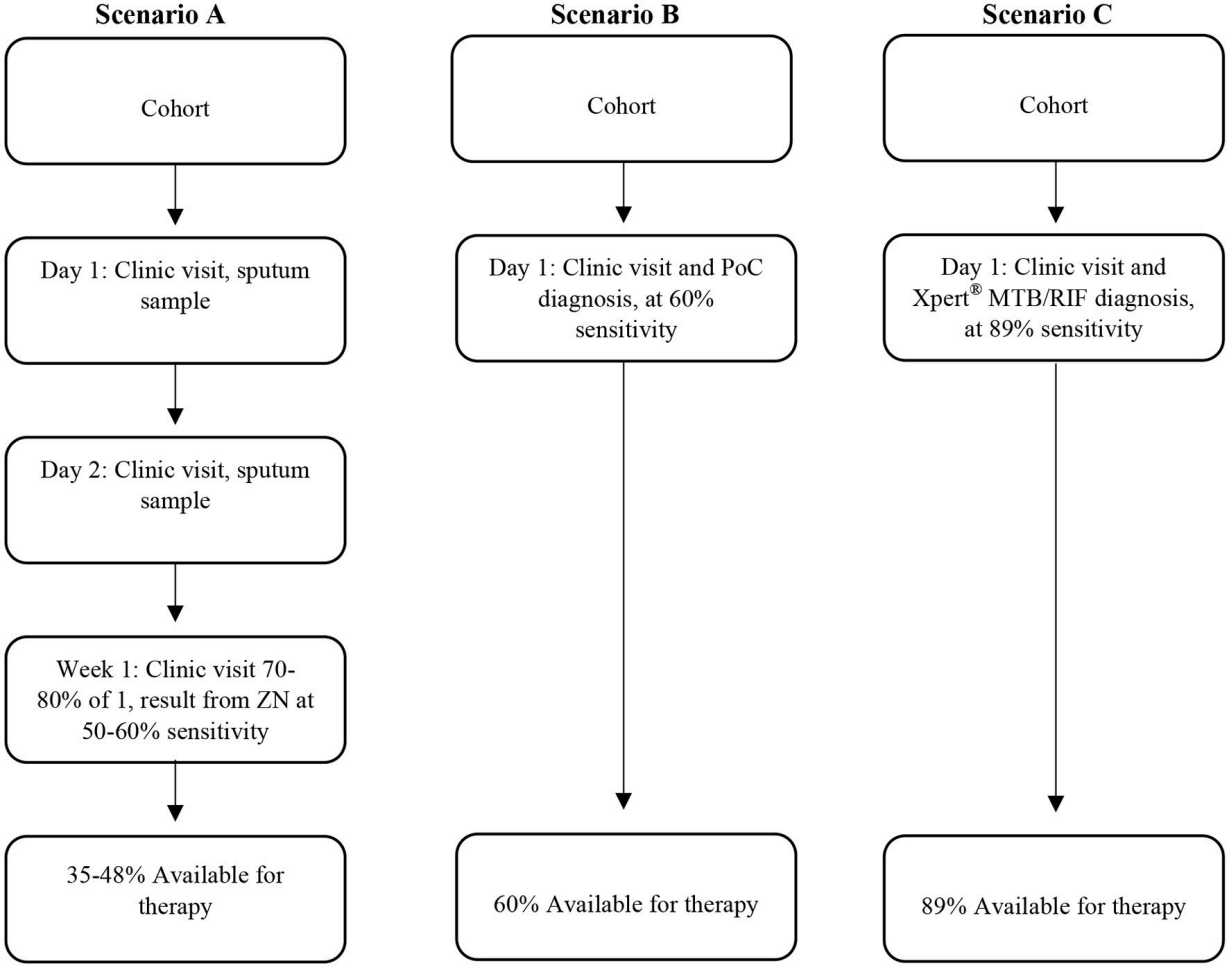
Different scenarios representing different human TB diagnostic approaches which include the sensitivity of the diagnostic tests and corresponding availability of therapy for individuals. Scenario 1 is a previous TB program now obsolete, scenario 2 is hypothetical, illustrating the advantage of point of care test, and scenario 3 is what could be achieved using the GeneXpert system if used for same day diagnosis in the clinic.

One of the key elements envisaged for successful TB control remains the goal of a point-of-care (PoC) diagnostic test, the value of which is illustrated by scenarios (Figure 1): we highlight firstly the South African human TB diagnostic program prior to 2011, which required three sputum samples from a client on different days over a week. This resulted in a loss to follow up of 17–25% (9, 10). Let us also assume that we use the test still used in many resource-poor settings, i.e., acid-fast staining with diagnostic sensitivity of 50–60%. The implication (Figure 1 scenario A) is that only a small percentage of patients initiated proper therapy, which allowed ongoing disease and transmission events (8). In a different hypothetical scenario (scenario B), using a test of the same sensitivity but PoC based, with immediate initiation of therapy, the proportion of TB cases that could initiate therapy almost doubles. Scenario B will also imply a reduction in infectiousness time and fewer transmission events. In a third hypothetical scenario (scenario C), an Xpert® MTB/RIF test is conducted (PoC) where indicated, therapy can be initiated immediately. Given the test sensitivity of 82–89% (11, 12), it implies that over 80% of TB cases could initiate therapy. Ignoring specificity discussion to illustrate this point, a high sensitivity PoC diagnostic test results in less loss or default.

By far the majority of the human TB diagnostic tests based on GeneXpert, are done at no cost to clients utilizing public clinics, since laboratory-based tests are done by the National Health Laboratory Service (NHLS, funded by the National Department of Health) which has many laboratories scattered in a network across the country (8). In contrast, the Department of Agriculture, Forestry and Fisheries (DAFF) subsidizes laboratory diagnostics at only one laboratory for BTB in suspect animal cases, but does not pay costs in full. Tests require that samples be taken at necropsy, or that fresh blood samples for immunological tests arrive within hours under ideal conditions, the latter being largely impossible in a large country with distant rural farms. Owners are not compensated for their animals which will result in a reluctance to test animals. Samples from necropsy are set up for mycobacterial culture followed by speciation (13). Unlike the NHLS, there is only one state lab, Onderstepoort Veterinary Institute (OVI), accredited for testing for bovine TB, largely because there is no financial incentive for other laboratories to be accredited. Such a monopoly is unlikely to be the best way forward.

Clearly, surveillance or suspicion of bovine TB should not lead immediately to slaughter and necropsy. Therefore, non-lethal diagnostics for animals are needed. Only once such diagnostics strongly suggest bovine TB, necropsy, culture, and speciation is done to confirm bovine TB. Bovine TB has been tested for in Bovidae by skin testing and more recently by *in vitro* blood-based interferon gamma (IFN-γ) release assays (IGRA) or other biomarkers (14–19). These tests although useful, are limited owing to the need for blood transport to accredited laboratories under time and temperature constraints, as well as the need for a reasonably well-equipped laboratory. In order to circumvent this logistics problem, serum-based diagnostics are being researched. Serum-based biomarker research in humans shows promise for a diagnostic, but as yet, although sensitivity is high (94%), specificity (73%) is inadequate for implementation (20). However, it may be that such biomarkers discovered for human TB diagnosis, may be applicable to bovine TB.

Bovine TB can also infect many species other than the Bovidae. Therefore, particularly in the case of wildlife, species-specific diagnostic tests may be required. This is necessary to prevent the disease from being maintained in an ecosystem outside of monitored hosts, e.g., cattle or buffaloes and where there may be concerns for endangered species, such as rhinoceroses. Failure to diagnose and treat a TB case has significant downstream cost implications, not least of which is ongoing transmission and disease propagation. Thus, a considerable and ongoing investment in the best diagnostics and control programs to implement these is justified.

## Retention

Many TB control programs suffer client losses along the care cascade. Such work shows the importance and advantages of the “Holy Grail” of TB researchers, the PoC diagnostic (21). The consequence of losses on the cascade is that successful completion of treatment for TB was estimated to be only 53% of cases (8). In the case of livestock or wildlife, the difficulties involved in accessing animals for repeat testing or dealing with positive responders are familiar to state veterinarians. No similar quantitative care cascade loss studies have been done in veterinary medicine in South Africa and thus information is anecdotal. However, the future cost of missed cases, as for humans, cannot be overemphasized.

## Treatment

There is perhaps little that can be learnt from current therapeutic management of human TB and extrapolated to animals. The standard treatment for TB in humans is antibiotic therapy (22), which with the exception of animals in captivity is not feasible in animals. Sometimes physical isolation is also practiced, i.e., the TB case is placed in a treatment facility to isolate them from the general populace. For TB in animals, the same basic principle applies: remove the bacterial threat by removing the animal (i.e., physical isolation), usually by slaughter.

## Adherence

The basic clinical principle applies: complete the course of treatment. This must apply, whether it is antibiotic treatment in humans, movement control or removal of animals with TB, usually by slaughter. Failure to do so will result in ongoing disease and transmission, and failure to eradicate the problem (22).

## Follow-up

This is an important step and often not done in human TB management in higher incidence areas owing to sheer volume of work and resource limitations. The reason for this activity is that even under ideal conditions and with proper adherence, some individuals will experience recurrent disease. Furthermore, prior to becoming bacillus negative, TB cases can transmit the disease. Ideally, therefore, treated and cured individuals need follow up for at least 2 years (23) and their contacts should be investigated. In the case of free-living humans, particularly in a high incidence society, investigating all contacts is impossible. Likewise for free-ranging wildlife. However, these principles are part of bovine TB control practice in South Africa, i.e., test and remove and subsequent follow up testing and retesting until disease is cleared according to protocol. This practice should always be followed. It is encouraging that even culling of limited infected animals in a free-ranging wildlife system can reduce prevalence rate (7).

Although the steps discussed above are arguably critical for TB control, there are many other factors that are important and will impact on any control measures undertaken. Some of these are discussed further below.

## Transmission

Arguably the most important step in combatting TB is to stop transmission. Close contact is important, but not definitive for transmission. For example, a study in a very high incidence area showed that only a small proportion of human TB cases result from household contact (24, 25). Furthermore, the passive detection of TB cases in high prevalence communities is insufficient to limit disease transmission (8, 26). We still have an inadequate understanding of TB transmission, although we know that aerosol transmission is one of the main sources for humans, and most likely also bovis. In the case of some other animals, it may be ingestion of contaminated meat or biting. Clearly, adequate distance must be maintained to avoid ongoing transmission. Therefore, attention should be given to the potential for a contaminated environment, and there should be space and free airflow such that transmission may be minimized.

## Infection, latency, and disease

It is generally stated that (in the absence of immunosuppression), only 10% of infected humans will develop active TB (4, 27). Traditionally and commonly stated: approximately half of those who will develop active disease will do so within 2 years after infection and the other half sometime after that, owing to reactivation of latent infection (LTBI) (28). Controversy characterizes opinions concerning whether a positive diagnostic assay, such as those that are host-based, really prove disease or are indicative of infection but do not necessarily represent disease or the presence of live bacilli. We previously considered four possible states: (1) not exposed, (2) exposed and infected, no response detectable, no sign of disease, (3) infected, bacilli present, no active disease (latent TB), (4) infected, active disease. In clinical medicine, distinguishing between these four states is not necessarily clear. A recent comprehensive review (23), suggests that the burden of disease from latent TB in humans has been vastly overestimated, suggests only three states and that TB has a shorter incubation period than previously thought. If this is correct, it has major implications for public health. Unfortunately, there is little clear-cut data on whether three or four states apply to the multiple animal hosts of *M. bovis*, nor clear-data regarding progression between states.

Therefore, the interpretation of immunological tests for human as well as bovine TB is complex. Possible outcomes of exposure from cattle to *M. bovis* are believed to be in line with that of humans. Briefly, following exposure to bacilli, the innate immune response can either clear the infection or fail to do so. This failure then leads to the need for intervention by the host's adaptive immune response. A successful response leads to the clearance of the infection with no delayed-type hypersensitivity responses (skin test and whole blood gamma interferon release assay negativity), or failure leads to active disease (skin test and IFN-γ release assay positivity) (29). In cattle, failure to detect visible lesions at *post-mortem* examinations does not indicate absence of infection (30). A systematic review of many studies has previously shown that 50% of reactor animals had no visible lesions (31), which was seen in a separate study where only 43% of reactors had visible lesions at slaughter (32). This suggests that as for humans (23) active disease may be significantly underestimated in studies where culture is the gold standard.

Recent modeling suggests that the WHO's (human) TB elimination target cannot be achieved by 2050 using LTBI screening as the sole control strategy (33, 34). The assumptions used include maximum coverage, no imported infections due to travel and migration, and application of an additional 4% annual decrease. This model suggests that a TB incidence of <1/100,000 will only be achieved about 50 years after implementation of LTBI screening and prophylactic treatment (33). These findings are optimistic assumptions, but illustrate the difficulties involved in eliminating TB when LTBI exists. Furthermore, they emphasize that continued surveillance and follow up will be essential. However, if latent TB is far less important than previously assumed, then eradication or good control far sooner than this is possible. Therefore, in veterinary medicine, the approach taken thus far has been wise, i.e., if any test is positive, take action. This should arguably continue to be the case and is probably the reason for the low prevalence of bovine TB in domestic stock in South Africa.

## Bovine TB in wildlife

Although bovine TB in livestock appears to be of low prevalence in South Africa (Table 1), this is not the case in at least three of our large national park systems (35, 36). Thus, far no effective plan has been made to combat it in an open system in South Africa, although some limited culling has been done in one park (7). Such areas pose a risk for spread beyond the park boundaries, but is limited as far as possible by testing, animal movement control, and breeding of disease-free animals, such as TB free herds of African buffalo (37). Insufficient research has been done to show whether or not this disease will impact species to affect the ecosystem and which species are maintenance or end-stage hosts.

## Economics

Stable systems require a healthy society, a healthy economy and a healthy environment. TB, whether in animal or human form impacts on all three of these pillars. The problem with giving inadequate attention to current TB using as the excuse “we can't afford it,” will leave us with the situation we currently have. The latest estimates (2014) from WHO are that 1.7 billion humans were latently infected by *M. tuberculosis* (28). We have no idea how many animals are infected by *M. bovis*, as a comparison, but an estimated 147,000 human (zoonotic) cases of bovine TB alone per annum occur (38). This implies many animal cases and neglect now will mean high future costs.

## Way forward

The nature of TB, whether human or animal form, makes eradication in the short term impossible. However, it is clear that transmission must be stopped in order to eradicate the disease. The essential lessons from this are many: one cannot be complacent, one cannot relax vigilance, and care for this disease (39). Active and latent cases must be dealt with before eradication can be considered.

Countries or regions should take the threat of bovine TB seriously. If this is not the case, then perhaps we can learn from one initiative started in South Africa recently to try to improve TB control. A TB Think Tank was established (40) bringing together researchers in the basic sciences, clinical sciences, epidemiology, social sciences, public health, and Health systems experts, and government staff. This body has promoted evidence-based decision-making, and in addition, lobbied successfully for increased funding for TB management (human) in South Africa. By involving national TB control staff and other experts, it is believed that significant impact on TB can be achieved. Similar think tank initiatives could be developed for other settings including bovine TB control to support evidence-based policy development and disease control and lobby for the finances to support such efforts.

## Author contributions

All authors listed have made a substantial, direct and intellectual contribution to the work, and approved it for publication.

### Conflict of interest statement

The authors declare that the research was conducted in the absence of any commercial or financial relationships that could be construed as a potential conflict of interest.
